# A Notable Address on State Medicine

**Published:** 1900-09

**Authors:** 


### THE TEXAS MEDICAL JOURNAL.

AUSTIN, TEXAS.

A MONTHLY JOURNAL OF MEDICINE AND SURGERY.

EDITED AND PUBLISHED BY

F. E. DANIEL, M. D., AND S. E. HUDSON, M. D.

Published Monthly at Austin, Texas, by Drs. Daniel and Hudson. Subscription price $1.00 a year in advance.

Eastern Representative: John Guy Monihan, St. Paul Building, 220 Broadway, New York City.

Official organ of the West Texas Medical Association, the Houston District Medical Association, the Austin District Medical Society, the Brazos Valley Medical Association, the Galveston County Medical Society, and several others.

### A Notable Address on State Medicine.

The *Denver Medical Times,* that excellent up-to-date periodical, edited by Dr. Thomas Hayden Hawkins, gives in full in the August issue the address on State Medicine by Dr. Wm. P. Munn, of Denver, delivered before the Colorado Medical Society June 20, 1900. It occupies thirty pages of the journal, and has marginal notes for ready reference to the various subjects treated. It is a masterly paper, and would make an excellent "campaign document" in the effort to enlighten the public on the subject of State medicine and public hygiene. A copy of it should be in the hands of every legislator in the land. While the paper deals for the first ten pages or so with the subject in a general way, giving in a clear manner an outline of what is embraced in the terms, the greater part is devoted to a review and criticism of Governor Thomas' most remarkable veto message to the Colorado legislature, vetoing the Medical Practice Act, in which he sets forth his reasons for his very remarkable action. Governor Thomas has been roundly abused for his action, but we have not before seen a calm and dispassionate review of the subject. Dr. Munn's criticism, while dispassionate and dignified, is, nevertheless, a most thorough dissection, showing the fallacy of the governor's position and the sophistry of his argument. Governor Thomas seems to not have comprehended the main essentials of the subject, and harps continually on interference with personal rights; that is, "the State shall not interfere with the citizen in his right to choose his own physician." Such a thing was never contemplated by the bill, nor by any bill. The object of the bill, and of all such bills, is to make it impossible for the citizen to select an incompetent, and therefore dangerous, physician; to see that he has no class to select from except those who have given the State evidence of their qualifications to be entrusted with the privilege of practicing medicine. The people cannot discriminate between the competent and the incompetent. The object of the bill is to remove the incompetent from the field. A man, the father of a family, might be so imbued with the doctrine of "personal rights" as to leave his children free to select their own food, for instance, but he should see that nothing of a deadly or dangerous nature is included in the lot from which to select, they being in ignorance of the danger. This is a homely simile, but of practical application. As to the alleged injustice of throwing out of practice many who cannot come up to the contemplated standard of requirements to be adopted in the proposed examinations, it is very remarkable that the governor, a great lawyer, does not appear to know that such legislation as he was vetoing could not affect any person who had at the time of the passage of the act qualified as a physician under any existing law or any law previously existing. The TEXAS MEDICAL JOURNAL commends the address of Dr. Munn to every physician who has a pride in his profession, and who favors reform in the great abuse of the practice of medicine. Doubtless Editor Hawkins or Dr. Munn would be pleased to send a copy free upon request to any of our readers. We regret that the length of the address precludes the possibility of our reproducing it entire. We quote below, however, a portion of it, bearing on "personal rights," etc. The bill is almost identical with that proposed by our own Texas State Medical Association, so summarily disposed of last session.

Dr. Munn said:

"The veto message makes the same assumption that is so often made by citizens when they reach the judicial bench. These individuals assume that doctors may not be entrusted with the exercise of discretion in behalf of the public, while all other classes of citizens, and lawyers especially, are to be so trusted. Three lawyers are appointed by a political friend to act as judges, let us say, of some appellate court. The appointments are made in acknowledgment of political services rendered. Life, liberty, property rights, human happiness, public welfare are all entrusted to these men, and there seems no impropriety in two out of three finally deciding such important matters. It makes no difference that the methods by which they arrive at a decision are as incomprehensible to the average sensible man as the movements of a jelly fish; the individuals so entrusted with authority are lawyers, turned into infallible judges by the magic touch of the executive, and can therefore do no wrong. But three doctors, selected from a profession which we hope is at least as reputable as that of the law; three men of decent, well ordered lives; three men who love their fellows and have devoted their services to the well being of the race; three such men are not to be entrusted with the responsibility of deciding upon the proper exercise of a public function by persons of whose professed attainments they are especially competent to judge. Oh, most sapient veto; oh, most cogent cozener of muddy and muddled brain cells; oh, most supreme example of superb self-satisfaction, temporarily enthroned in place of power; if this argument of thine shall carry weight in uniform application to all branches of government, then legislatures, courts and councils shall pass together into outer darkness, anarchy shall reign and all the functions of government cease. Yea, even the power of veto might then be relinquished by the all-wise and considerate one, in momentary fear that he perhaps would err in its exercise.

"The veto further says: 'The sum of all experience is progress, and the public health is benefited precisely as sanitary laws are observed, investigation of disease and remedies promoted and men and women left free to choose their own physicians.'

"Here is as bland, as intricate and as deceptive a statement as was ever framed by any platform orator; the ready wit of the special pleader shows in its construction; three truths are presented in consecutive order, and then an untruth by implication is added so deftly, so naturally, with such a show of fairness, as to almost deceive the 'elect,' which is the favorite term of the veto in referring to the regular medical profession. On the platform such a specious verbal deception might readily pass uncaught and uncorrected; but when committed to cold type, and read carefully after a year's interval, it is at once seen that the three preceding and undeniable statements neither lead up to nor can by any process of mental gymnastics be connected with the final statement as to 'men and women being left free to choose their own physicians.'

"The implication that the proposed law, either directly or indirectly, assumed to take away such freedom of choice from any man or woman, or that it authorized the prosecution, conviction or punishment of any one exercising such freedom of choice, finds no basis in the bill itself. Not one word or one sentence could ever be so construed by any sane person; the veto quotes no sentence that could be so interpreted. The bill would have punished the charlatans and law-breakers, not their victims; and it was the charlatans and law-breakers, not their victims, who originated and first presented the argument which the veto here puts forth. These charlatans have already been described in a prior paragraph of the message as 'loud in pretense and reckless in the use of remedies, taking advantage of the afflicted by giving assurance to their hopes, only to rob them of health and substance.'

"Yet notwithstanding this scathing arraignment, the veto proceeds to adopt their arguments and to plead their cause in eloquent and misleading phraseology. It presents in more respectable garb the arguments that they originated; it shows that they ought not to be amenable to any law because laws are difficult to enforce; it pleads that guilt be its own punishment and that the law shall utter no condemnation; it asks that charlatanry be not only tolerated but respected. Its sympathy for them finally rises to such a pitch that it joins with them in throwing mud at all who refuse to accept them as professional associates. There is an old adage recently revived whose homely words seem to apply in this connection: 'One cannot lie down with dogs and rise up without fleas.'"

